# A Case Report of Follicular Lymphoma in a Crohn’s Disease Patient Treated With Azathioprine

**DOI:** 10.7759/cureus.50839

**Published:** 2023-12-20

**Authors:** Nadeer Kottavadakkeel, Reefat Farzina, Arun Rajaram, Sathia Mannath, Abida Sunil

**Affiliations:** 1 Gastroenterology, Pilgrim Hospital Boston, United Lincolnshire Hospital National Health Services (NHS) Trust, Lincolnshire, GBR; 2 Gastroenterology and Hepatology, Aster Medcity, Kochi, IND; 3 Otolaryngology, Pilgrim Hospital Boston, United Lincolnshire Hospital National Health Services (NHS) Trust, Lincolnshire, GBR; 4 Diabetes and Endocrinology, Pilgrim Hospital Boston, United Lincolnshire Hospital National Health Services (NHS) Trust, Lincolnshire, GBR

**Keywords:** immunomodulators, inflammatory bowel disease, paraspinal mass, cauda equina syndrome, follicular lymphoma

## Abstract

Inflammatory bowel disease (IBD) is an autoimmune disorder marked by chronic inflammation affecting the intestines. Crohn's disease (CD) and ulcerative colitis (UC) fall under the IBD umbrella, necessitating diverse treatments, including steroids, immunomodulators like 6-mercaptopurine (6-MP) and azathioprine (AZA), and biological agents. The prolonged use of immunomodulators, such as AZA, is associated with an elevated risk of developing lymphomas. This case report centers on a 77-year-old gentleman regularly monitored by Gastroenterology, undergoing long-term AZA therapy for CD management. He presented with palate swelling and acute-on-chronic back pain, diagnosed with follicular lymphoma in the palate with metastasis. Palliative radiotherapy was administered for the paraspinal lesion, and the patient is currently stable. In conclusion, this case underscores the importance of recognizing the heightened risk of neoplasms, especially lymphomas, in patients undergoing prolonged immunomodulator therapy. It emphasizes the need for a vigilant and comprehensive approach to patient care, transcending conventional paradigms.

## Introduction

Inflammatory bowel disease (IBD) is an autoimmune disorder affecting the intestine, characterized by chronic inflammation. Crohn's disease (CD) and ulcerative colitis (UC) are encompassed within IBD, and their treatment frequently involves various agents, including steroids, immunomodulators such as 6-mercaptopurine (6-MP) and azathioprine (AZA), and biological agents. AZA is a thiopurine and a purine antimetabolite. Upon metabolism, it converts to 6-MP and functions by inhibiting the synthesis of DNA, RNA, and proteins, thereby impeding mitosis. It modulates the immune response by acting as an immunomodulator, immunosuppressant, or disease-modifying antirheumatic drug (DMARD). AZA/6-MP is commonly used as a first-line immunomodulator for maintaining disease remission in IBD. Common side effects of thiopurine therapy include allergic reactions (resulting in skin rashes), gastrointestinal complaints, hair loss, pancreatitis, hepatitis, and bone marrow suppression (leading to pancytopenia). Prolonged treatment duration has been controversial due to uncertainties regarding the risk of inducing diseases like intestinal and extra-intestinal malignancies [1]. The International Agency for Research on Cancer (IARC) has classified AZA as a carcinogen. While most population-based studies show no inherent association between IBD and an increased risk of lymphoma [2,3], in patients undergoing long-term AZA or 6-MP therapy, the risk of lymphoma increases fourfold compared to the general population [4].

This report presents an unusual case of a 77-year-old gentleman who had been on maintenance AZA for over 20 years. He developed lymphoma in the palate and paraspinal area, resulting in palatal swelling and acute spinal cord compression.

## Case presentation

A 77-year-old man, diagnosed with systemic hypertension, coronary artery disease post-coronary artery bypass grafting (CABG) in 2018, degenerative spinal disease, and CD since 1977 (on regular olsalazine and AZA for 15 years), presented to his general practitioner in August 2022 with a painless palate lump noticed three months prior. He was promptly referred under the two-week wait cancer pathway to the Oro-Maxillofacial surgeon (OMF). A former smoker who quit 50 years ago, he's been under regular follow-ups in Gastroenterology clinics for CD. Occasional flare-ups were well managed with IV and oral steroids, and steroid enemas. His recent colonoscopy indicated quiescent proctosigmoiditis. Despite multiple attempts to discontinue AZA, his symptoms worsened. Finally, in August 2022, AZA was ceased, and he was maintained on oral olsalazine.

**Figure 1:**
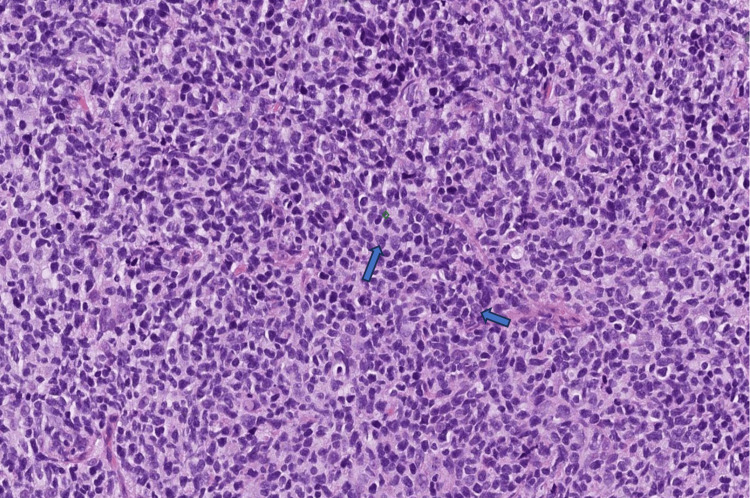
Biopsy specimen of the right palatal lesion in 20X This shows a dense monotonous infiltrate composed predominantly of enlarged lymphoid cells with irregular/cleaved nuclei typical of centrocytes (the main component of low-grade follicular lymphomas).

**Figure 2:**
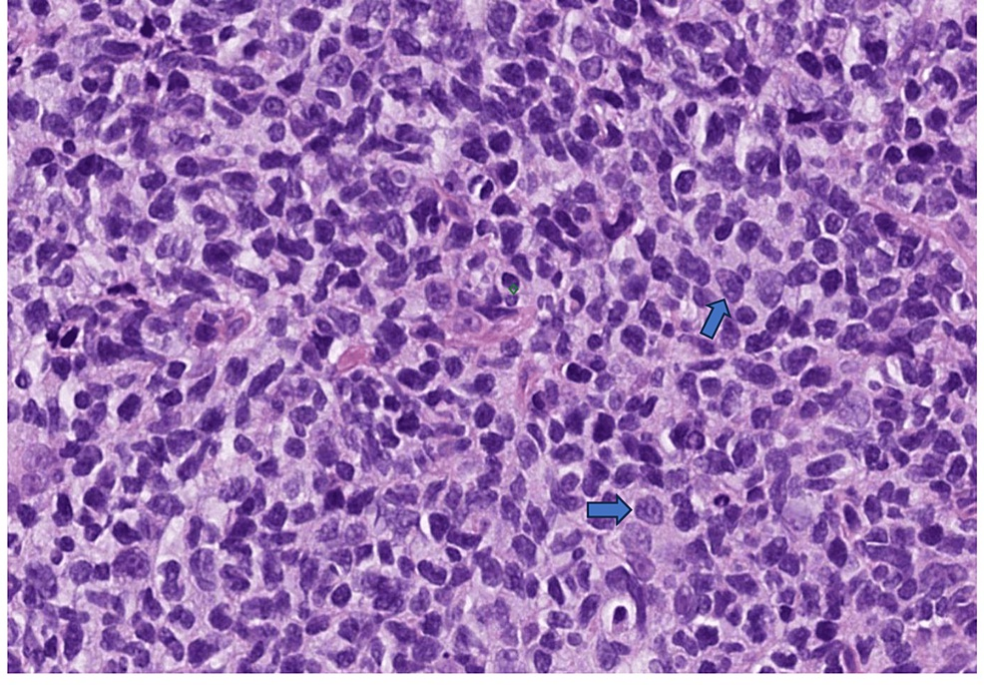
Biopsy specimen of the right palatal lesion in 40X This shows a dense monotonous infiltrate composed predominantly of enlarged lymphoid cells with irregular/cleaved nuclei typical of centrocytes (the main component of low-grade follicular lymphomas).

The OMF surgeon observed a 2 cm painless soft lump on the hard palate, suspecting a probable hard palate neoplasm. An urgent fine needle aspiration (FNA) and contrast MRI of the neck were arranged. Routine blood tests, including cell counts and CRP levels, were within normal limits. The magnetic resonance imaging (MRI) revealed an enhancing lesion on the right hard palate extending into the maxillary alveolus, likely a minor salivary gland tumor, and a small enhancing lesion on the left hard palate. FNA cytology was paucicellular and inconclusive, prompting a repeat biopsy. The subsequent incisional biopsy confirmed follicular lymphoma (FL), grade 1-2 (Figures 1-2). The OMF surgeon recommended a hematology opinion after reviewing scans and biopsy results.

The hematologist reviewed the patient, ordering a positron emission tomography and computed tomography (PET-CT) scan, and bone marrow biopsy for staging. A lymphoma multidisciplinary team (MDT) meeting was arranged to discuss the results. The September 2022 bone marrow biopsy revealed normocellular findings with no evidence of lymphoma. A PET-CT in October 2022 was performed, but before the scan results were available, he experienced acute worsening of lower back pain with paresthesia of lower limbs, leading to admission from the Accident & Emergency (A&E).

Upon admission, routine blood tests revealed leucocytosis with neutrophilia and lymphocytopenia (Table 1). Serum lactate dehydrogenase levels were elevated at 390 U/L (140-280 U/L).

**Table 1:** Routine blood test results

Investigation	Result	Range
C-reactive protein (CRP)	1.1	0-5 mg/L
Full blood count		
Haemoglobin	149	132-170 g/L
White cell count	14.9	4.3-11.2 g/L
Red cell count	4.90	4.29-5.69 x 10^12/L
Haematocrit	0.429	0.387-0.492
Mean corpuscular haemoglobin (MCH)	30.4	26.9-33 pg
Mean corpuscular haemoglobin concentration (MCHC)	347	320-359 g/L
Neutrophils	14.21	2.1-7.4 x 10^9/L
Lymphocytes	0.57	1.0-3.6 x 10^9/L
Monocytes	0.06	0.3-1.0 x 10^9/L
Eosinophils	0.00	0.02-0.5 x 10^9/L
Basophils	0.01	0.02-0.1 x 10^9/L
Electrolytes		
Magnesium	0.79	0.70-1.0 mmol/L
Sodium	136	133-146 mmol/L
Potassium	4.3	3.5-5.3 mmol/L
Urea	6.4	2.5-78 mmol/L
Creatinine	59	59-104 umol/L
Glomerular filtration rate (GFR)	>90	90-200 mL/min
Bone Profile		
Calcium	2.35	2.2-26.6 mmol/L
Adjusted Calcium	2.42	2.20-2.60 mmol/L
Phosphate	1.33	0.80-1.50 mmol/L
Liver Function Tests		
Bilirubin	6	0-21 umol/L
Alanine transaminase (ALT)	19	0-41 U/L
Alkaline phosphatase (ALP)	70	30-130 U/L
Total Protein	62	60-80 g/L
Albumin	35	35-50 g/L
Globulin	27	20-40 g/L
Clotting Profile		
Prothrombin time (PT)	12.2	10.0-13.2 s
International normalised ratio (INR)	1.0	0.9-1.2 1/1
Activated partial thromboplastin clotting time (APTT)	30.7	28.3-35.0 s

**Figure 3:**
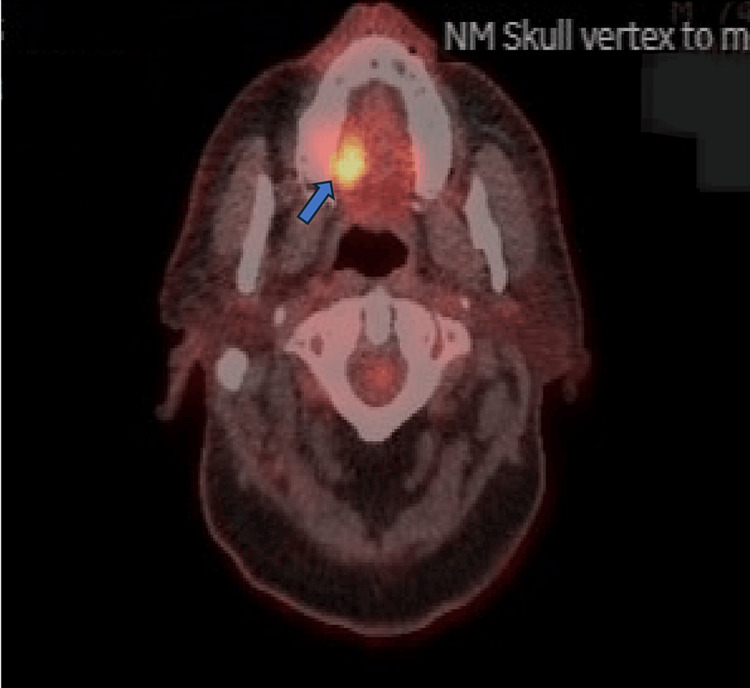
FDG avid lesion in the palate FDG: 18F-fluorodeoxyglucose

**Figure 4:**
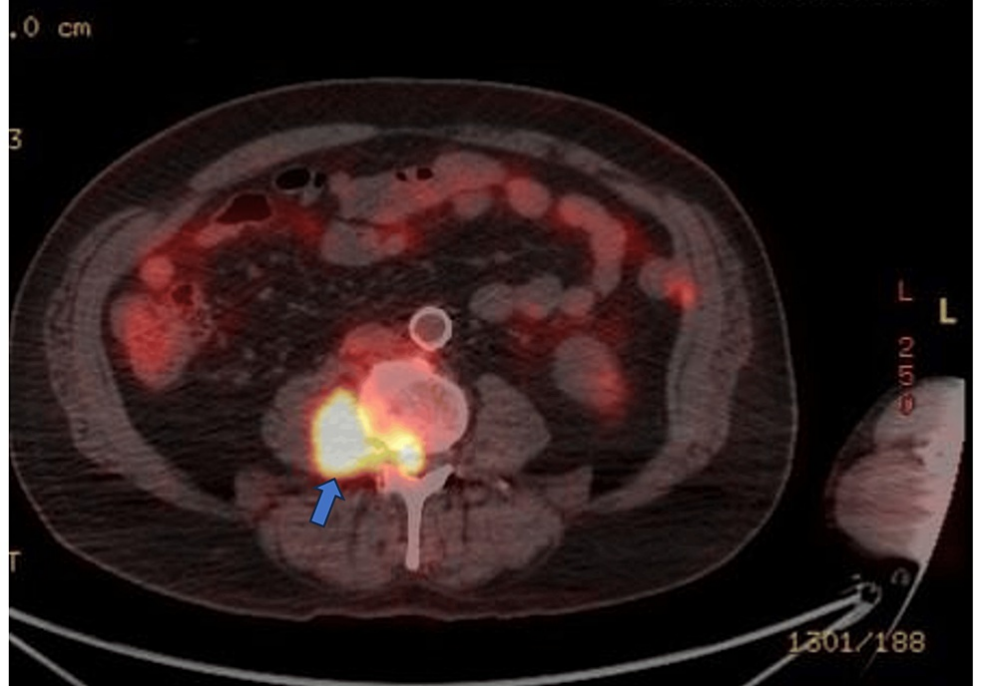
FDG avid paraspinal lesion extending to the spinal canal FDG: 18F-fluorodeoxyglucose

On the day of admission in October 2022, the previously done PET-CT scan was reported. It revealed 18F-fluorodeoxyglucose (FDG) avidity in the palate (Figure 3), paraspinal area at the level of L3 vertebrae extending into the spinal canal (Figure 4), and retroperitoneal lymph node involvement. Left inguinal nodal disease was also noted, indicating multifocal lymphomatous metabolic stage IV disease. The report indicated that paraspinal disease could potentially have neurological consequences, prompting a suggested MRI.

**Figure 5:**
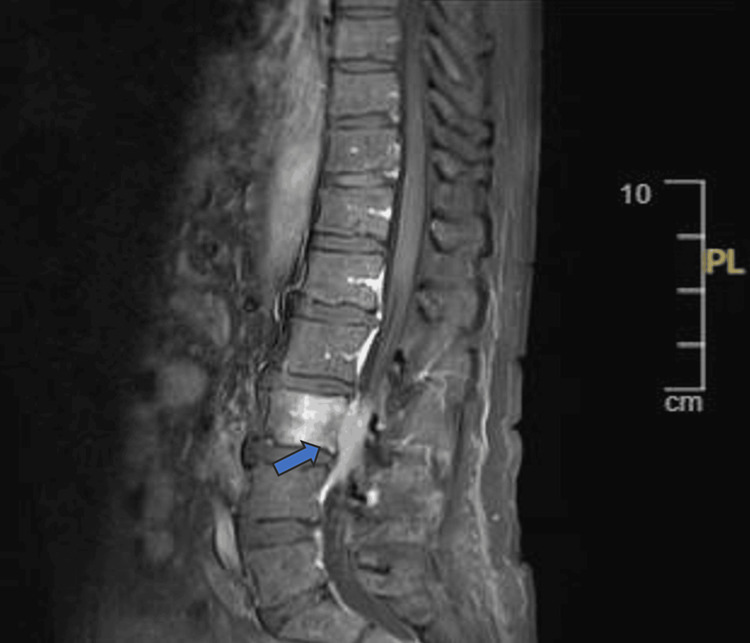
Marrow involvement of L3 with para vertebral and epidural soft tissue components Focal marrow involvement of L3 with para vertebral and epidural soft tissue components, leading to spinal canal narrowing and compression of cauda equina nerve roots.

Given the PET-CT report and his presentation with features suggesting cord compression, an urgent MRI of the whole spine was conducted. The results confirmed focal marrow involvement of L3 with para vertebral and epidural soft tissue components, leading to spinal canal narrowing and compression of cauda equina nerve roots (Figure 5).

In light of the clinical presentation and MRI findings, the suspicion of Cauda Equina Syndrome arose, leading to an urgent referral to the neurospinal team at a nearby specialist center. They recommended mobilizing the patient as tolerated and indicated that they would provide further guidance after conducting an urgent MDT. The lymphoma MDT recommended by Hematology was conducted. A plan was formulated to obtain a CT-guided biopsy, lumbar puncture, CSF study, flow cytometry of CSF, and an oncology referral for radiotherapy. He was commenced on high-dose steroids, and the Oncology team suggested palliative radiotherapy for the paraspinal lesion following the biopsy. CT-guided biopsy of the right paraspinal mass, CSF study, and blood immunophenotyping were conducted. After a week, he was discharged to another hospital within the Trust to undergo radiotherapy.

The spine surgery team in the new hospital opted against active intervention and recommended proceeding with palliative radiotherapy. He began palliative radiotherapy for his spinal lesion, completing a total of 12 cycles in November 2022. The CSF study conducted before the initiation of radiotherapy revealed no signs of infection or malignancy. Blood immunophenotyping indicated no signs of leukemia. The CT-guided biopsy from the paraspinal mass confirmed FL of grades 1-2. A review of lymphoma MDT was held and the plan was established to finalize the radiotherapy and conduct a repeat PET-CT three months after radiotherapy to monitor progress.

In December 2022, the patient observed an enlargement of the soft palate lesion. Consequently, an early PET-CT was arranged, revealing persistent uptake in the paravertebral lesion extending to the spinal canal, along with sustained uptake in the right palate and left inguinal node. Following the lymphoma MDT review, the recommendation was to persist with conservative management. Palliative chemotherapy with O-CVP (obinutuzumab, cyclophosphamide, vincristine, prednisolone) was suggested only if symptoms worsened. In March 2023, the Hematologist conducted another review, and the patient was clinically well with no B symptoms of lymphoma noted. A repeat PET-CT was performed, as scheduled after radiotherapy, revealing the resolution of lymphoma in the spine but persistent FDG avidity in the palate and left groin. The Hematologist recommended no further intervention, considering the patient's improved clinical condition with the absence of B symptoms. In July 2023, he underwent a review in the Gastroenterology clinic and was advised to continue taking olsalazine. Further follow-up with an IBD nurse was recommended. He continues to have regular follow-ups with Hematology for his underlying FL disease.

## Discussion

A recently published Scandinavian cohort study reveals a growing risk of lymphoma over time in individuals with CD, contrasting with the absence of such an increase in those with UC. The study indicates that the hazard ratio for lymphoma has risen over the past two decades, aligning with the increased use of immunomodulators and biologics during the same period. Additionally, the research identifies an elevated risk of aggressive B-cell non-Hodgkin's lymphoma (NHL) in both CD and UC patients. Notably, CD patients also exhibit a heightened risk of T-cell NHL. The highest risks are observed in individuals undergoing combined therapy (immunomodulators and biologics) or second-line biologics. Importantly, the study also detects increased risks in patients who had not been exposed to these drugs before [5].

When not exposed to anti-tumor necrosis factor (TNF) and/or thiopurine, the overall risk of lymphoma in IBD patients remains relatively low and appears comparable to that of the general population [2]. Notably, factors such as advanced age (>65 years), male gender, and the use of thiopurine have been identified as significant contributors to an elevated risk of incident lymphoma [6]. Specifically, patients with IBD under thiopurine treatment, including AZA or 6-MP, exhibit a statistically significant increase in lymphoma risk [6-8]. Recent case reports have drawn attention to the occurrence of lymphoma in patients with IBD undergoing immunomodulator or anti-TNF therapy. An illustrative example is the occurrence of primary hepatic lymphoma in a patient with CD receiving thiopurine and anti-TNF therapy. These instances are highlighted in recently published case reports, contributing to our understanding of this association [9].

Our case underscores the uncommon diagnosis and presentation of FL, a form of NHL, in a CD patient undergoing long-term AZA therapy.

The CD is a chronic inflammatory condition affecting the gastrointestinal tract, marked by persistent inflammation that can extend throughout various parts of the digestive system. This inflammation may lead to complications such as fistulas, abscesses, strictures, and the development of malignancies. The range of malignancies associated with CD encompasses both those affecting the gastrointestinal tract and those occurring outside the digestive system [10]. According to a meta-analysis from 2009, which explored the risk of extraintestinal malignancies in individuals with IBD, it was determined that, overall, there was no heightened risk of extraintestinal cancers among IBD patients. However, site-specific analyses revealed that CD patients had an increased risk of cancer of the upper gastrointestinal tract, lung, urinary bladder, and skin. Patients with UC had a significantly increased risk of liver-biliary cancer and leukemia but a decreased risk of pulmonary cancer [11].

Immunomodulators and anti-TNF agents have been proven to be effective in the treatment of IBD and can have a significant positive impact on the lives of these patients. Immunosuppressors are part of the mainstay of treatment of IBD. One of which is thiopurine. The risk of malignancy during thiopurine therapy is associated with the cumulative dose and duration of AZA administered [12], levels of thiopurine metabolites, and mutations in thiopurine methyltransferase [13].

Approximately 30% of lymphomas associated with AZA use manifest in the gastrointestinal tract. While Hodgkin's lymphomas often present as mediastinal masses, the presentation of NHL varies widely, with estimates of gastrointestinal involvement ranging from 5% to as high as 60% [14]. Reports indicate a higher incidence of lymphomas in the intestinal tract among individuals with IBD [15,16]. The elevated risk of lymphoma in IBD patients is significantly lower than that observed in organ transplantation, a condition associated with much higher levels of immunosuppression [17].

Among the lymphomas, NHL emerged as the predominant histological subtype (83.9%). Notably, diffuse large B-cell lymphoma (DLBCL) and FL stood out as the most prevalent NHL subtypes in IBD patients, constituting 30% and 13% of cases, respectively. In comparison to de novo lymphoma, primary intestinal lymphoma (PIL) constituted a significant proportion of lymphomas in IBD patients, ranging from 22% to 75%, while mucosa-associated lymphoid tissue (MALT) lymphoma was relatively underrepresented. Epstein-Barr virus (EBV) positivity was noted in a substantial proportion of tumors, ranging from 44% to 75%. Survival outcomes for lymphoma in IBD patients were comparable to those observed in de novo lymphoma [18].

In contrast to findings from specialized centers, population-based studies reveal a diminished yet statistically significant increase in lymphoma risk in individuals with IBD using thiopurines. This increased risk does not appear to endure following the cessation of therapy. Individuals aged over 50 exhibit the highest absolute risk of lymphoma per year while on thiopurines, with men under 35 also potentially constituting a high-risk demographic. It is imperative to weigh the risks of lymphoma against the potential therapeutic benefits for all IBD patients [9].

The management of FL depends on the disease stage. In stage 1 lymphomas, radiation therapy is the favored option for grades 1, 2, and 3a lymphomas. On the other hand, individuals with grade 3b FL undergo treatment with intensive regimens (like R-CHOP) utilized for other aggressive lymphomas (e.g., DLBCL). Managing stage II-IV FL primarily aims at enhancing quality of life, relieving symptoms, and addressing cytopenias. Asymptomatic patients are typically monitored closely without any active intervention. Anti-CD20 antibodies (obinutuzumab, rituximab) are combined with chemotherapy regimens for addressing symptomatic advanced FLs. Autologous hematopoietic stem cell transplantation is the preferred approach for recurrent/relapsed patients or those with transformed higher-grade lymphoma [19].

Despite increasing evidence supporting the safety and efficacy of immunomodulator therapy for IBD, anxiety about developing NHL remains a prevalent concern for both patients and physicians when deciding to use these medications for IBD treatment [20]. Nevertheless, considering the scale of the association, even if the heightened risk is entirely linked to the medications, it is unlikely to outweigh the potential benefits of these medications for the majority of patients.

## Conclusions

AZA, employed as immunomodulator therapy in IBD management, poses a heightened risk of lymphoma development, albeit rarely. Prolonged AZA use can lead to FL. Despite this, the benefits of AZA outweigh the risks, making it a preferable option compared to alternatives.
